# Interferon-independent processes constrain measles virus cell-to-cell spread in primary human airway epithelial cells

**DOI:** 10.1128/spectrum.01361-23

**Published:** 2023-09-19

**Authors:** Lorellin A. Durnell, Camilla E. Hippee, Roberto Cattaneo, Jennifer A. Bartlett, Brajesh K. Singh, Patrick L. Sinn

**Affiliations:** 1 Department of Microbiology and Immunology, Carver College of Medicine, The University of Iowa, Iowa City, Iowa, USA; 2 Department of Molecular Medicine, Mayo Clinic, Rochester, Minnesota, USA; 3 Stead Family Department of Pediatrics, Carver College of Medicine, The University of Iowa, Iowa City, Iowa, USA; Thomas Jefferson University, Philadelphia, Pennsylvania, USA

**Keywords:** contagion, cell-associated virus, innate immunity, morbillivirus, paramyxoviridae

## Abstract

Amplification of measles virus (MeV) in human airway epithelia may contribute to its extremely high contagious nature. We use well-differentiated primary cultures of human airway epithelial cells (HAE) to model *ex vivo* how MeV spreads in human airways. In HAE, MeV spreads cell-to-cell for 3–5 days, but then, infectious center growth is arrested. What stops MeV spread in HAE is not understood, but interferon (IFN) is known to slow MeV spread in other *in vitro* and *in vivo* models. Here, we assessed the role of type I and type III IFN in arresting MeV spread in HAE. The addition of IFN-β or IFN-λ1 to the medium of infected HAE slowed MeV infectious center growth, but when IFN receptor signaling was blocked, infectious center size was not affected. In contrast, blocking type-I IFN receptor signaling enhanced respiratory syncytial virus spread. HAE were also infected with MeV mutants defective for the V protein. The V protein has been demonstrated to interact with both MDA5 and STAT2 to inhibit activation of innate immunity; however, innate immune reactions were unexpectedly muted against the V-defective MeV in HAE. Minimal innate immunity activation was confirmed by deep sequencing, quantitative RT-PCR, and single-cell RNA-seq analyses of the transcription of IFN and IFN-stimulated genes. We conclude that in HAE, IFN-signaling can contribute to slowing infectious center growth; however, IFN-independent processes are most important for limiting cell-to-cell spread.

**Importance:**

Fundamental biological questions remain about the highly contagious measles virus (MeV). MeV amplifies within airway epithelial cells before spreading to the next host. This final step likely contributes to the ability of MeV to spread host-to-host. Over the course of 3–5 days post-infection of airway epithelial cells, MeV spreads directly cell-to-cell and forms infectious centers. Infectious center formation is unique to MeV. In this study, we show that interferon (IFN) signaling does not explain why MeV cell-to-cell spread is ultimately impeded within the cell layer. The ability of MeV to spread cell-to-cell in airway cells without appreciable IFN induction may contribute to its highly contagious nature. This study contributes to the understanding of a significant global health concern by demonstrating that infectious center formation occurs independent of the simplest explanation for limiting viral transmission within a host.

## INTRODUCTION

Measles virus (MeV) is a highly contagious human respiratory virus (1). Its replication strategy differs from that of other respiratory viruses because a lymphatic phase precedes the respiratory phase (2). Initially, cells that express the signaling lymphocytic activation molecule (SLAM), such as dendritic cells, collect MeV from the airways and ferry it through the epithelial barrier (3, 4). These myeloid cells then spread the infection to other SLAM-expressing cells, including lymphocytes, in the local lymph nodes and in primary immune tissues (5, 6). Massive MeV replication in immune tissue depletes B and T lymphocytes and leads to long-term immunosuppression (7 – 10). Finally, infected immune cells expressing the MeV glycoproteins on their surface deliver multiple MeV genomes *en bloc* to nectin-4-expressing airway epithelial cells (11 – 14).

To characterize cellular mechanisms that control MeV infection of airway epithelia, we rely on well-differentiated primary human airway epithelial cells (HAE) that mimic the variety and morphology of many surface cell types observed in human airways *in vivo*. Although HAE lack the immune cell component of the MeV life cycle, this model has been used extensively to study innate immune responses to viral, bacterial, and fungal pathogens (15 – 18) as well as asthma and cystic fibrosis disease states (19 – 21). Typical MeV infection of permissive immortalized cells results in syncytia destined for cell death (22). However, MeV infection of HAE results in the formation of infectious centers that differ from syncytia. The defining features of an infectious center include intact plasma membranes (although some cytoplasmic sharing is observed), nuclei remain separated, and minimal cytopathic effect (12, 23). The number of infected cells in an infectious center is highly variable and can range from ~10 to 1,000 cells but typically average ~200 cells. MeV spreads rapidly for 3 or 5 days, after which cell-to-cell spread stops and infectious center growth plateaus. What stops the cell-to-cell spread of MeV in HAE is not yet understood.

In many viral infections, an effective innate antiviral response includes interferon (IFN) induction by pattern recognition receptors (24, 25). Indeed, IFN induction has been documented in different MeV infection models (26 – 30). In SLAM-expressing immune cells (i.e., activated B cells, T cells, dendritic cells, and macrophages), type I IFNs (IFN-α and IFN-β) establish the antiviral state (31). Immune-activated airway epithelial cells express little IFN-α but abundant IFN-β as well as type III interferon, IFN-λ (32, 33). IFN-λ mediates the antiviral response against respiratory viruses including pneumoviruses, paramyxoviruses, rhinoviruses, and coronaviruses (34, 35). In response to MeV infection, IFN-λ is produced in dendritic cells (31) and detected in the lung lavage of experimentally infected monkeys (36, 37).

HAE retain the functional IFN signaling cascades that robustly induce type I and III IFNs *in vivo* in response to viral infections and have been used to model innate immune responses to viral infections, including respiratory syncytial virus (RSV), pneumovirus (38, 39), influenza A virus (40), and SARS-CoV-2 (41), as well as other *Paramyxoviridae* family members such as Nipah virus (42) and parainfluenza viruses (43). Of note, RSV-infected HAE show increased antiviral cytokine and chemokine expression despite minimal induction of type I IFNs (38, 39). Here, we assessed the contribution of IFN to restricting MeV cell-to-cell spread. We contrast the effects of IFN on MeV and RSV infection, another non-segmented negative-strand RNA virus with a different spreading strategy based on apical budding and re-entry (38, 44). We conclude that, unlike RSV, MeV does not induce a discernable IFN response in HAE, recapitulating *in vivo* results in experimentally infected non-human primates (36, 45). Thus, HAE rely on different processes to restrict the growth of these two viruses.

## RESULTS

### MeV spread in HAE plateaus and is non-cytopathic

**Fig 1:**
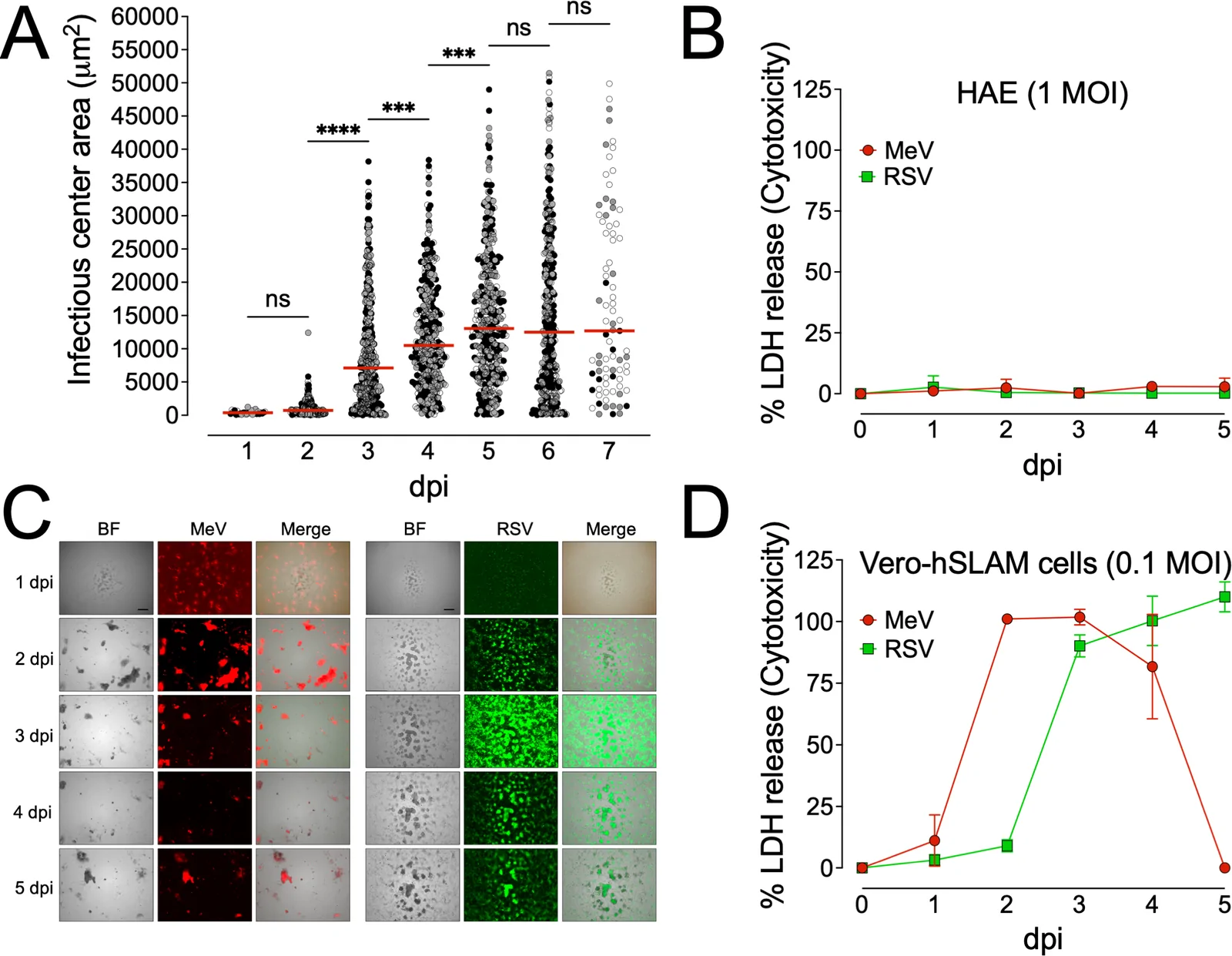
MeV spread in HAE plateaus and is non-cytopathic. HAE were infected basolaterally with MeV expressing mCherry upstream of N-protein (1 MOI) or apical with RSV expressing GFP upstream of N-protein (1 MOI). (**A**) Infectious center area was measured using ImageJ software. Each circle represents a single infectious center, and each color represents a donor. *n* = 3 donors; dpi, days post infection. All images were blinded in ImageJ prior to measurements using a blind-analysis tool. Measurements were unblinded just prior to GraphPad Prism input. Significance was determined by non-parametric Dunn’s multiple comparisons test. Red bars indicate median area. (**B**) LDH cytotoxicity levels from MeV- or RSV-infected HAE (1 MOI, 4 h) were measured in basolateral medium. *n* = 3 donors. Representative images of MeV- and RSV-infected (0.1 MOI) (**C**) Vero-hSLAM cells 1–5 dpi are shown. Infected cells were imaged on a Keyence fluorescence microscope. Brightfield (BF), reporter virus fluorescence, and merged images are shown. Scale bar = 3 mm. LDH cytotoxicity levels from the supernatants of MeV- or RSV (0.1 MeV)-infected Vero-hSLAM cells (**D**). Cells plated at 50,000 cells/well and infected for 2 h. *n* = 3 wells per virus infection. Data are plotted as mean ± SE.

Using a MeV (wild-type IC-323) that expresses mCherry fluorescent protein, we infected HAE from the basolateral surface and imaged infectious centers over a period of 5 days (Fig. S1A and B). Consistent with previous studies (12, 22, 23, 46), MeV infection of HAE resulted in visibly detectable infectious center formation at 2 days post infection (dpi) followed by near cessation of cell-to-cell spread by ~5 dpi (Fig. 1A). Between 6 and 7 dpi, infectious centers often dislodge from the epithelium (46), evident by a decrease in the number of infectious centers by 7 dpi. Based on a lactate dehydrogenase (LDH) release assay, cell death was not observed in MeV- or RSV-infected HAE (Fig. 1B) (44). We previously followed infectious center growth for up to 3 weeks without detectable loss of transepithelial electrical resistance and 2 weeks without detection of a caspase-3 apoptosis marker (46). In contrast to HAE, syncytia were observed 2 dpi in MeV- or RSV-infected Vero-hSLAM cells (Fig. 1C). LDH release in MeV-infected Vero-hSLAM cells peaked 3 dpi and declined precipitously by 5 dpi, indicating that most cells were dead by this time (Fig. 1D). Of note, the MOI for HAE was 10-fold higher than Vero-hSLAM cells. MeV-induced cell death was also observed in H358 (interferon competent) cells, albeit, to a lesser extent than Vero-hSLAM (interferon defective) cells (Fig. S2A and B). These results illustrate the unexplained and unique observation that MeV infection of HAE leads to distinct foci of infectious centers that plateau in size 3–5 dpi.

### MeV spread is sensitive to exogenous IFN

**Fig 2:**
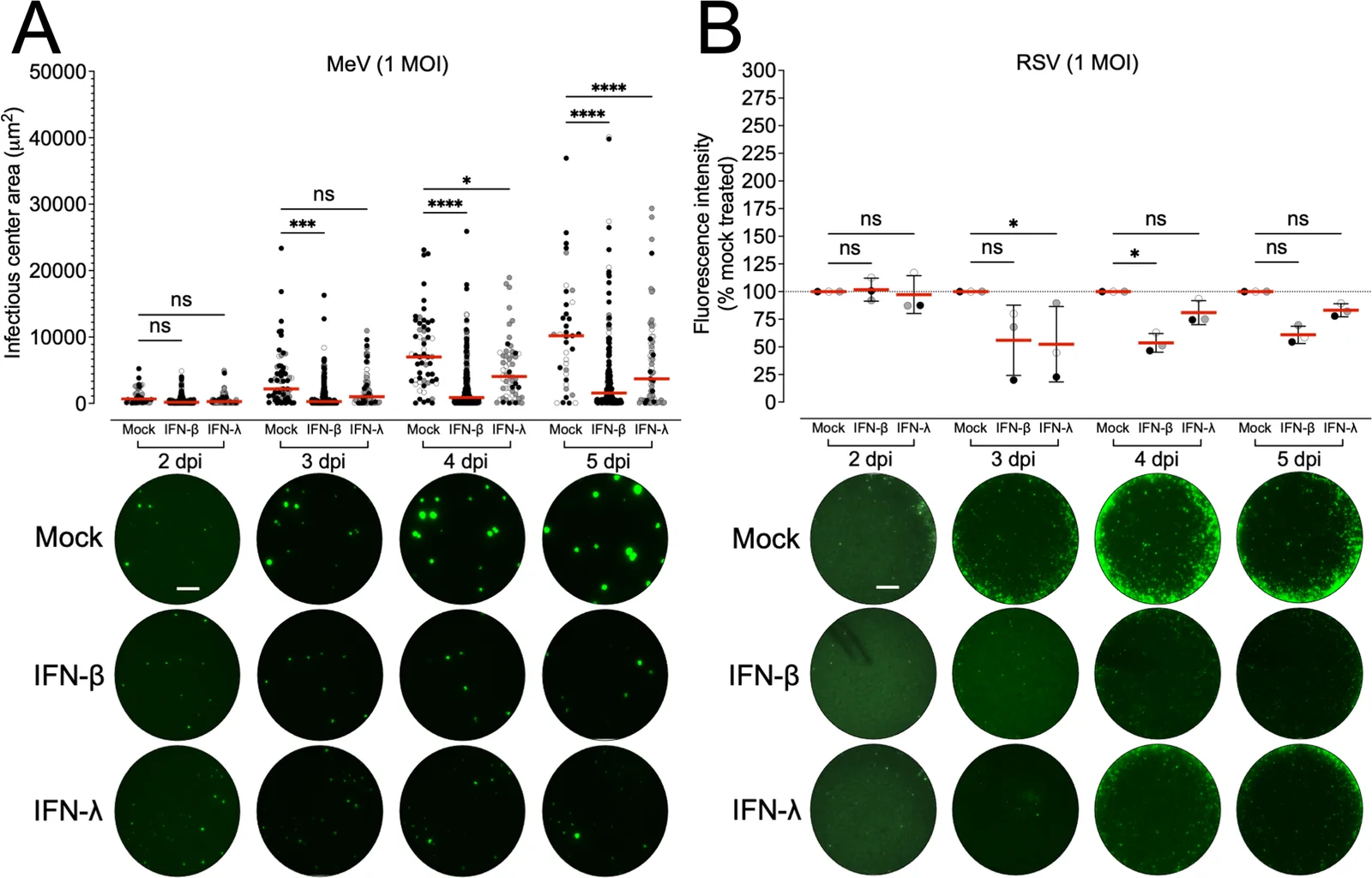
MeV is susceptible to exogenous IFN treatment. (**A**) HAE were infected at the basolateral surface with MeV expressing GFP upstream of N-protein (1 MOI) or (**B**) apical surface with RSV expressing GFP upstream of N-protein (1 MOI). Recombinant IFN-β (100 ng/mL), IFN-λ (2 µg/mL), or PBS vehicle (mock) were added to the apical surface of cultures at 18 hpi. Treatment lasted 24 h. Transwells were imaged 2–5 dpi. (**A**) Infectious center size or (**B**) fluorescence intensity was quantified using ImageJ software. Images were blinded in ImageJ prior to measurements using a blind-analysis tool. Measurements were unblinded just prior to GraphPad Prism input. *n* = 3 donors, indicated by color. Representative low power images for each treatment are shown. Scale bar = 500 µm. Significance was determined by one-way ANOVA with Tukey’s multiple comparison corrections.

We sought to understand why the rapid phase of cell-to-cell spread of MeV in HAE ends by 3–5 dpi. We hypothesized that spread stops due to IFN-induced innate immunity, specifically type I and III IFN production and signaling. To determine if cell-to-cell spread was sensitive to IFN, HAE were infected with MeV using the standard protocol. Cells were then treated with recombinant IFN-β, IFN-λ1, or vehicle (mock) 18 h post infection (hpi), once the initial infection was established. The HAE were imaged and the GFP+ area for every infectious center in the culture was quantified using ImageJ at indicated time points post infection (Fig. 2A). As compared to mock-treated cultures, treatment with IFN-β resulted in a 64%–69% decrease in infectious center area beginning at 3 dpi. Treatment with IFN-λ1 resulted in a 34%–48% decrease in infectious center area beginning at 3 dpi. In parallel, we infected HAE with RSV followed by IFN-β or IFN-λ1. Because RSV infection does not result in infectious center formation, the total fluorescence intensity of the culture was quantified. As shown, both IFN-β and IFN-λ1 post-treatment resulted in a ~50% decrease in fluorescence intensity beginning at 3 dpi (Fig. 2B). Thus, exogenously supplied type I or III IFN can reduce MeV spread in HAE.

### Blocking IFN does not significantly impact MeV cellular spread

**Fig 3:**
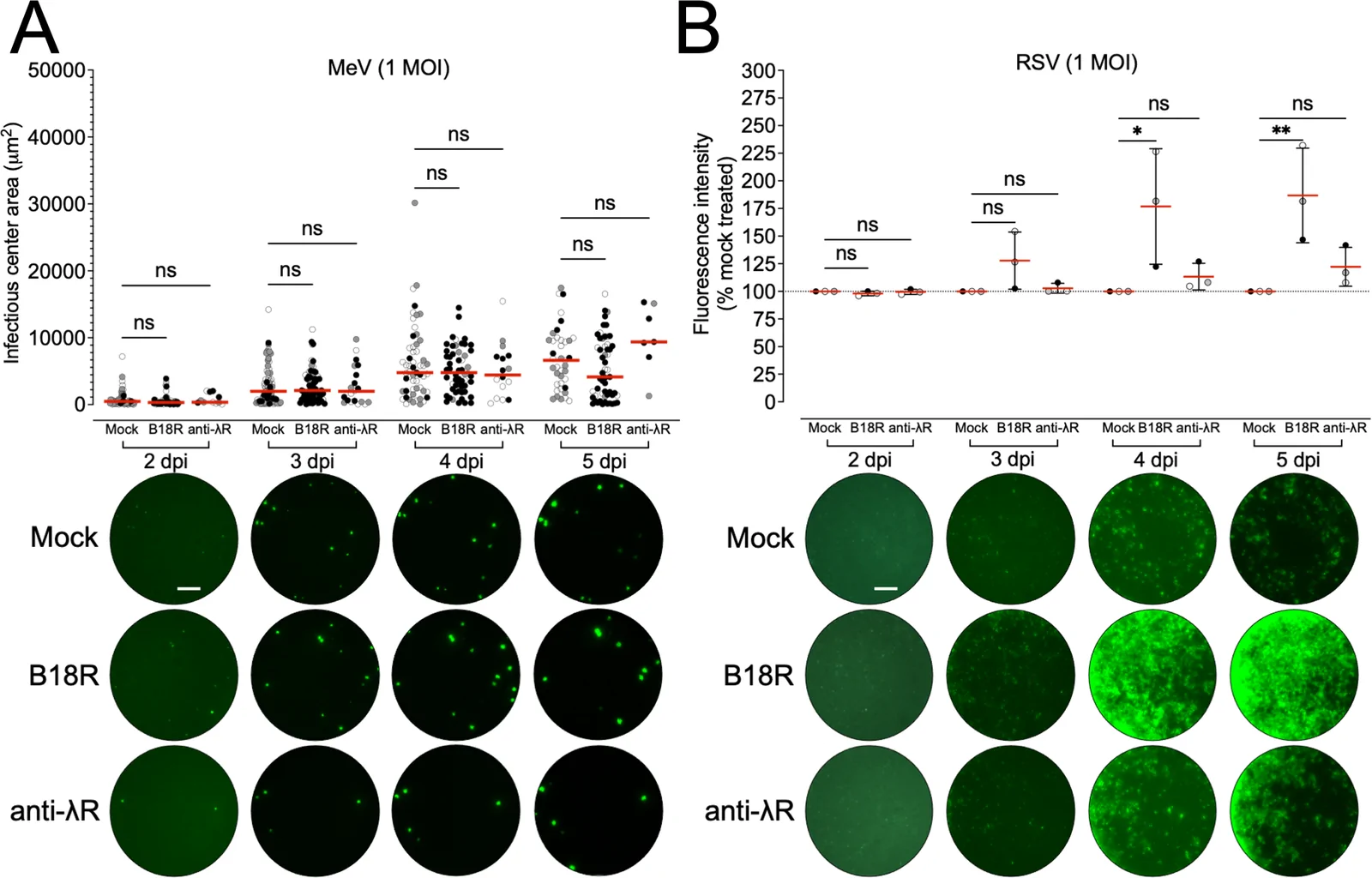
Blocking IFN in HAE does not increase IC size. (**A**) HAE were infected at the basolateral surface with MeV expressing GFP upstream of N-protein (1 MOI) or (**B**) apical surface with RSV expressing GFP upstream of N-protein (1 MOI). Recombinant B18R (2 µg/mL), anti-λ1 blocking antibody (50 ng/mL), or PBS vehicle (mock) were added to the apical surface of cultures immediately post infection. Treatment lasted 24 h. Transwells were imaged 2–5 dpi. Infectious center size (**A**) or fluorescence intensity (**B**) was quantified using ImageJ software. Images were blinded in ImageJ prior to measurements using a blind-analysis tool. Measurements were unblinded just prior to GraphPad Prism input. *n* = 3 donors, indicated by color. Representative low power images for each treatment are shown. Scale bar = 500 µm. Significance was determined by one-way ANOVA with Tukey’s multiple comparison corrections.

We next asked if blocking type I or III IFN signaling would lead to prolonged MeV cell-to-cell spread and subsequently increased infectious center area. Immediately after the standard 4 h MeV or RSV infection protocol, cultures were treated with a type I IFN decoy receptor (B18R), an anti-IFN-λ1 receptor-blocking antibody (anti-λR), or vehicle (mock). HAE were imaged and the GFP+ area for every infectious center in the culture was quantified (Fig. 3A). Interestingly, compared to mock-treated cultures, the median infectious center areas of the IFN-blocked cultures did not increase. In contrast, B18R treatment resulted in an expected increase in RSV-conferred fluorescence intensity, suggesting that type I IFN is a barrier to RSV spread in HAE (Fig. 3B). There was a small trend for increased fluorescent intensity following anti-λR treatment of RSV-infected HAE that did not reach statistical significance. Contrary to our hypothesis, this experiment provided a first indication that IFN may not be responsible for limiting MeV cell-to-cell spread in HAE.

### MeV lacking V-protein is not defective in HAE

**Fig 4:**
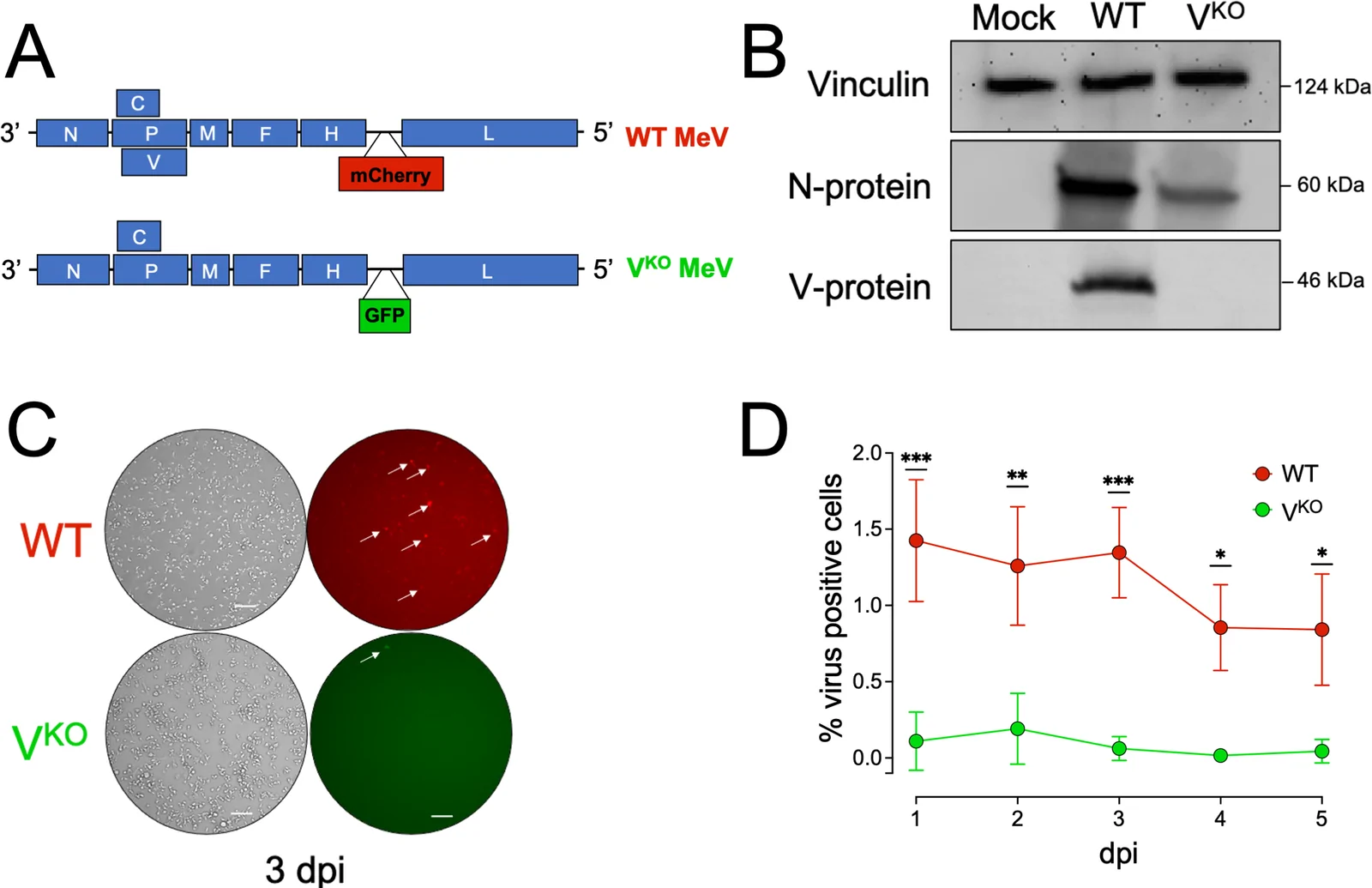
V^KO^ MeV is attenuated compared to WT MeV in macrophage-like cells. (**A**) Representative schematics of WT MeV and V^KO^ MeV. WT MeV expresses an mCherry cassette, and V^KO^ MeV expresses a GFP cassette between the hemagglutinin (**H**) and large (**L**) protein (polymerase). (**B**) Vero-hSLAM cells were infected with either WT MeV or V^KO^ MeV (1 MOI) for 2 h. Cell lysates were collected 2 days later, and western blot was used to detect N-protein, V-protein, or a vinculin control. (**C and D**) THP1 cells were infected (1 MOI) with either WT MeV or V^KO^ MeV for 4 h. (**C**) Cells were imaged at 3 days post infection (dpi). Arrows indicate infected cells. Scale bar = 500 µM. (**D**) Flow cytometry was used to quantify WT MeV (mCherry+) or V^KO^ MeV (GFP+) in THP1 cells daily for 5 dpi. Significance was determined by one-way ANOVA with Tukey’s multiple comparison corrections.

MeV lacking V-protein (V^KO^ MeV) (shown schematically, Fig. 4A) has a defective ability to antagonize the IFN host response, is attenuated *in vivo*, and is highly inflammatory in experimentally infected monkeys (47). We confirmed that V-protein expression was ablated in V^KO^ MeV-infected Vero-hSLAM cells via western blot (Fig. 4B). V^KO^ MeV was attenuated in TH1-derived macrophage-like cells as compared to WT MeV (Fig. 4C and D). As previously observed, V^KO^ MeV did not show a growth defect compared to WT MeV in Vero-hSLAM (Fig. S3A and B). At 3 dpi in IFN competent H358 cells, V^KO^ MeV was still more prevalent in the cell lysate than the cell supernatant, whereas WT MeV-infected cells were lysing by this time (Fig. S3C and D) (47).

**Fig 5:**
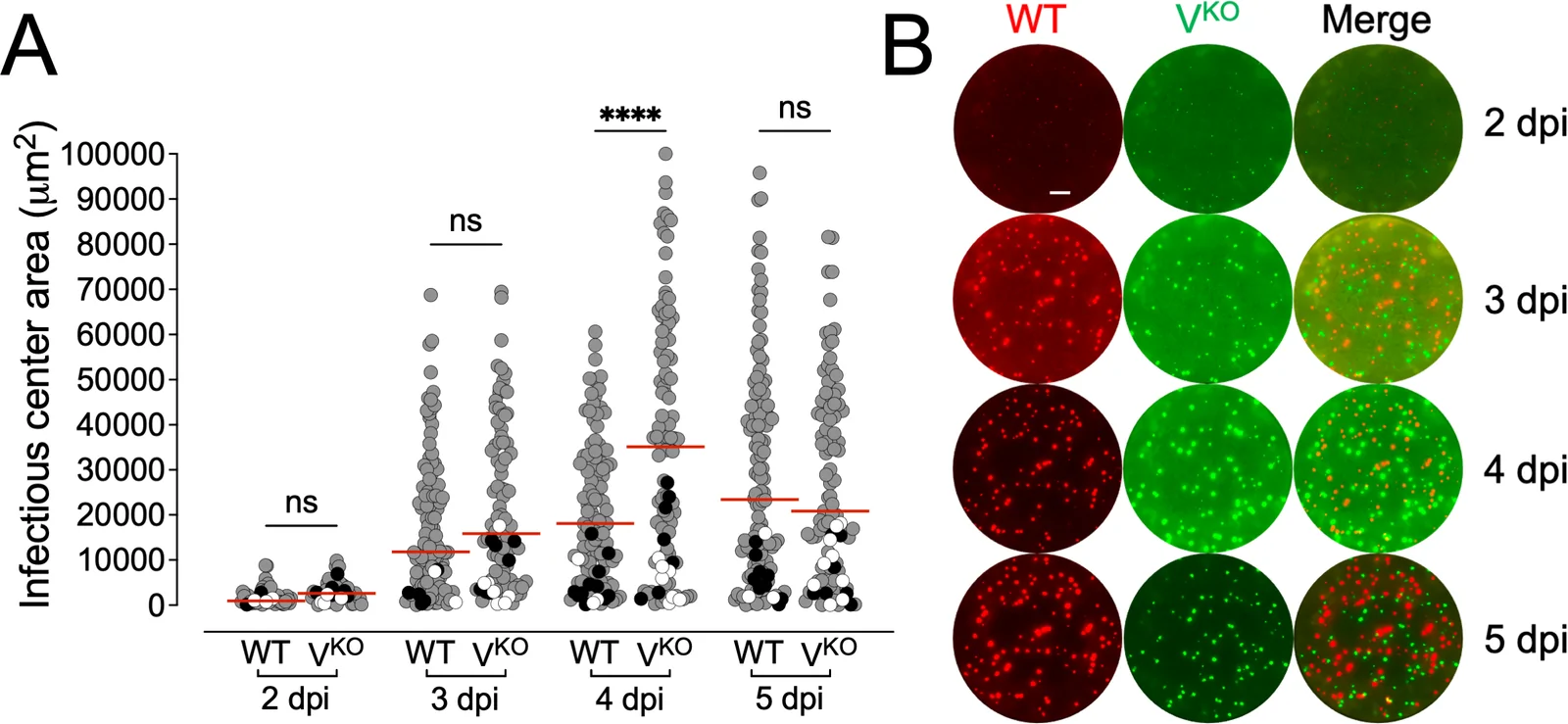
V^KO^ MeV is not attenuated in HAE. HAE were co-infected with WT MeV and V^KO^ MeV (1 MOI) for 4 h. Transwells were imaged 2–5 dpi. (**A**) WT MeV- and V^KO^ MeV-conferred infectious center size was assessed using ImageJ software. *n* = 3 donors, indicated by color. Significance was determined by one-way ANOVA with Tukey’s multiple comparison corrections. Representative low power images for each treatment are shown. (**B**) Representative images are shown. Scale bar = 500 µm.

To compare infectious center area over time in HAE, we co-infected with WT MeV (mCherry) and V^KO^ MeV (GFP). Co-infection avoids culture-to-culture variation. WT MeV and V^KO^ MeV infectious center areas were measured 2–5 dpi. Of note, V^KO^ MeV infectious center areas were not smaller than WT MeV infectious centers (Fig. 5A), and at 4 dpi, V^KO^ MeV infectious centers were larger than WT. Representative images are shown in Fig. 5B. To confirm that any differences in our ability to detect mCherry or GFP reporter proteins did not skew the area measurements, we used immunocytochemistry to detect N-protein of co-infected cultures. Infectious center size was first measured in N-protein+ cells. Following quantification, the mCherry or GFP status for each infectious center was revealed (Fig. S4A and B). These results recapitulated direct measurement of infectious center size using mCherry or GFP fluorescence. The results using V^KO^ MeV oppose the hypothesis that IFN limits cell-to-cell spread but are consistent with the results shown in Fig. 3.

A MeV lacking C-protein (C^KO^ MeV) is also available for study (47). C protein is key for minimizing replication mistakes, and its knockout results in the production of defective interfering RNA (48). Infection of HAE with C^KO^ MeV expressing GFP results in small and faint infectious centers (Fig. S5A and B). The C-protein may be involved in mitigating a cellular IFN response to MeV; however, the overall lack of detectable viral-expressed reporter fluorophore from the C^KO^ MeV in HAE prevented its inclusion in this study.

### MeV and MeV-V^KO^ infection results in minimal production and release of antiviral cytokines

**Fig 6:**
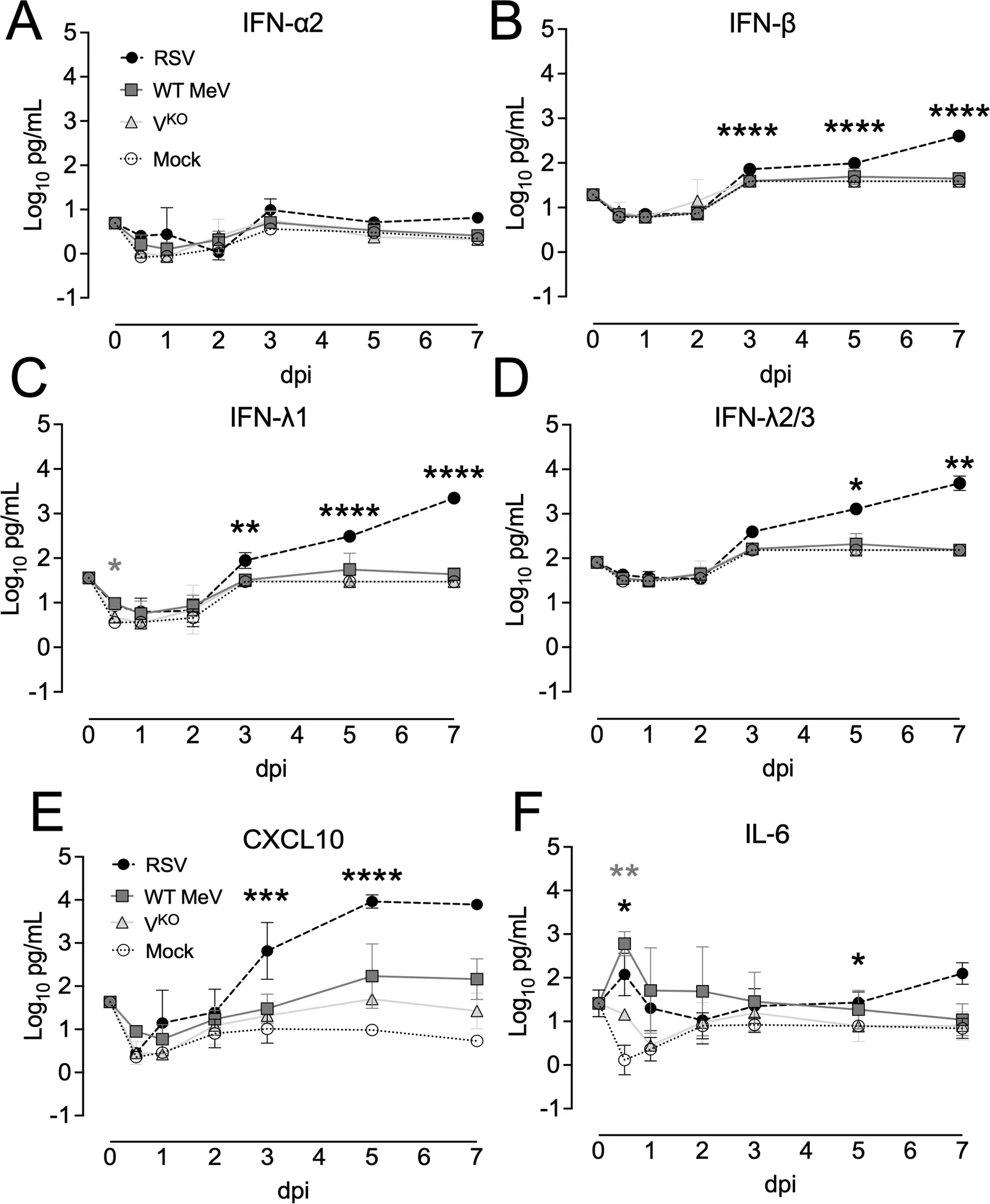
Neither WT MeV nor V^KO^ MeV induces basolateral release of IFN in HAE. HAE were either mock infected or infected with RSV, WT MeV, or V^KO^ MeV (1 MOI) for 4 h. Basolateral media were collected 12 hpi and everyday up to 7 dpi. Basolateral medium was replenished for each culture with fresh media after collection. Samples were analyzed for the presence of (**A**) IFN-α2, (**B**) IFN-β, (**C**) IFN-λ1, and (**D**) IFN-λ2/3. In addition, antiviral cytokines (**E**) CXCL10 and (**F**) IL-6 were analyzed using a human antiviral response panel. Significance was determined by one-way ANOVA with Tukey’s multiple comparison corrections.

To quantify the antiviral and proinflammatory cytokine protein release by MeV-infected HAE, we collected both apical washes and basolateral media from WT MeV, V^KO^ MeV, RSV, and mock-infected HAE cultures up to 7 dpi. A LEGENDplex Human Anti-Virus Response Panel (13-plex) cytokine screen was used to quantify apical or basolateral antiviral cytokine release. In basolateral media, IFN-α2 production was no different than mock for any virus (Fig. 6A). Of note, an initial drop at 0.5 day was observed for most proteins and likely the result of the initial fluid change. RSV infection resulted in a significant increase in IFN-β (Fig. 6B), IFN-λ1 (Fig. 6C), and IFN-λ2/3 (Fig. 6D), especially at later timepoints of infection. In contrast, WT MeV did not induce notable IFN over the mock condition, except for a small increase of IFN-λ1 at 12 hpi (Fig. 6C). V^KO^ MeV infection also resulted in little IFN. This was unexpected because V^KO^ MeV was shown to be inflammatory in macaques (47). In addition, IFN-α2, IFN-β, IFN-λ1, and IFN-λ2/3 protein levels following WT MeV or V^KO^ MeV infection were not elevated over the mock condition even after 5 days of accumulation without media changes (Fig. S6A). WT MeV did result in a modest increase in CXCL10 (Fig. 6E) and IL-6 (Fig. 6F) which are inflammatory cytokines previously reported to be present in plasma of MeV-infected individuals (49 – 51). However, no other cytokines in the LEGENDplex panel were detected in the basolateral media (Fig. S6B). We did not detect notable apical release of IFN or other inflammatory cytokines (Fig. S7).

### Type I and III IFN transcript levels are not elevated during MeV infection of HAE

**Fig 7:**
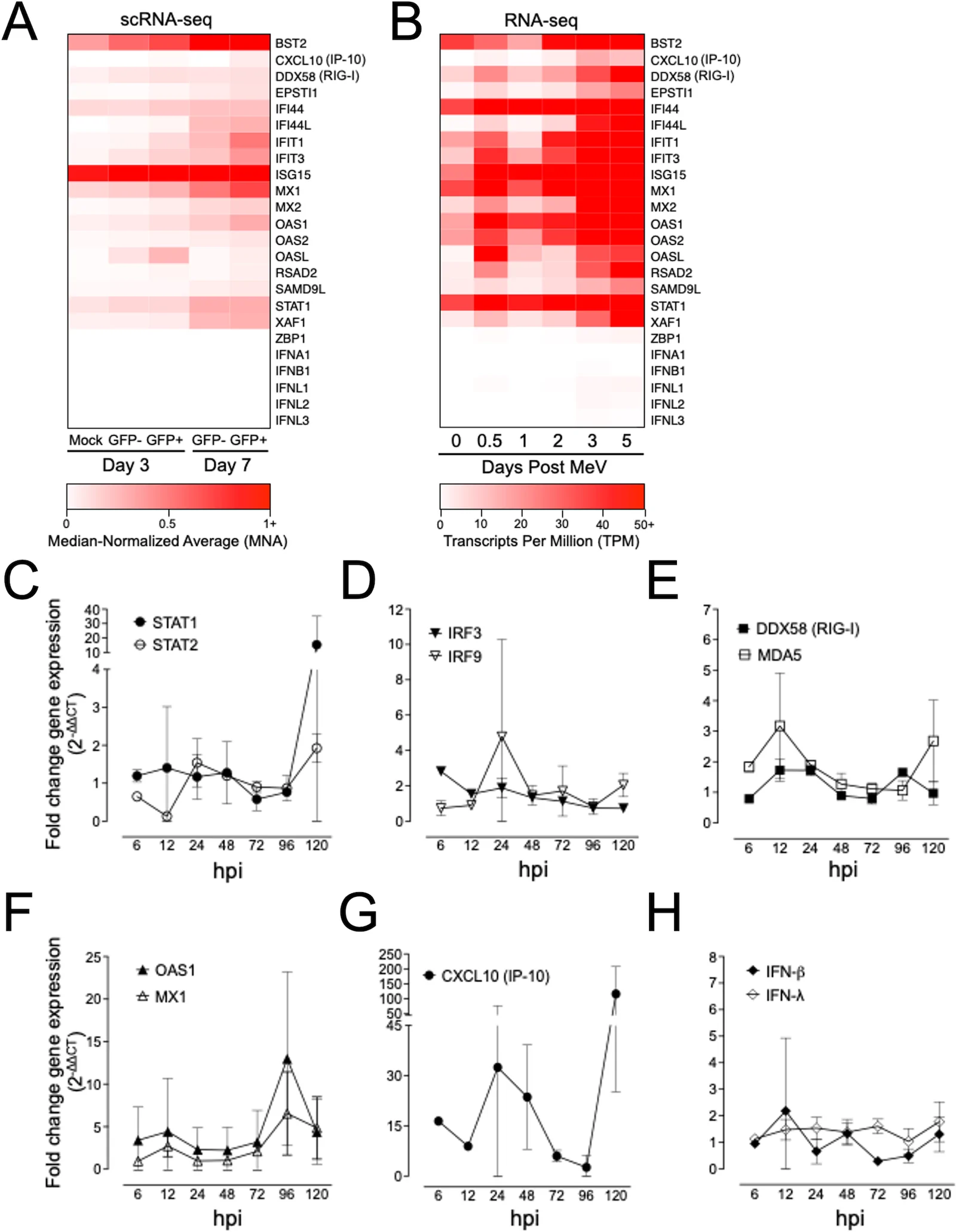
IFN RNA levels are not elevated during MeV infection of HAE. (**A**) Single-cell RNA sequencing was performed on mock or MeV-infected cultures 3 and 7 dpi. Each MeV-infected culture was FACS sorted into GFP+ or GFP− populations. Intensity shows measure of raw gene transcript counts normalized for the number of cells and total transcript reads in each group (MNA). Every condition included 10 pooled, matched donor HAE cultures, and sequencing was performed with a 10× Genomics scRNA-seq. (**B**) A heat map is shown depicting expression levels for selected innate immune genes measured by RNAseq of MeV-infected HAE. Transcript abundances are expressed as transcripts per million (TPM). MNA and TPM are both measures of raw transcript reads; however, direct comparison of the color intensity between the two heat maps is not necessarily meaningful. HAE were infected with MeV and collected for sequencing at 0, 0.5, 1, 2, 3, and 5 dpi. *n* = 4 donors. Heatmaps were constructed using RStudio. (**C–H**) qRT-PCR results of indicated genes from MeV-infected HAE 0.25–5 dpi. *n* = 3 donors.

Following MeV infection of HAE, we measured IFN mRNA abundance and mRNA abundance of known antiviral genes three ways: single-cell RNA sequencing (scRNA-seq), bulk mRNA next-generation sequencing (RNA-seq), and quantitative reverse transcriptase polymerase chain reaction (qRT-PCR) of selected genes. Our scRNA-seq data set of mock- or MeV-infected HAE cultures at 3 and 7 dpi was previously reported (46). Briefly, HAE were infected with a MeV that expresses GFP. Infected HAE cultures were enzymatically dissociated and then GFP+ and GFP− cells were isolated using fluorescence-activated cell sorting (FACS). Minimal IFNα, β1, λ1, λ2, or λ3 mRNA transcripts (Fig. 7A) or 16 other IFNs (Fig. S8A) were observed in MeV-infected cultures at 3 and 7 dpi. Similarly, RNA-seq of MeV-infected cultures at 1–5 dpi also failed to detect IFN mRNA (Fig. 7B; Fig. S8B). qRT-PCR indicated modest mRNA increases in mediators of the IFN signaling cascade, STAT1 and STAT2 (Fig. 7C), IRF3 and IRF9 (Fig. 7D), and the cytosolic RNA sensors, RIG-I and MDA5 (Fig. 7E). Two common antiviral genes, OAS1 and MX1, showed increased mRNA levels during the course of infection (Fig. 7A, B and F). As detected by qRT-PCR, CXCL10 mRNA increased in response to infection, consistent with the LEGENDplex screen (Fig. 6E and 7G). The lack of MeV-induced IFN mRNA was consistent between the scRNA-seq, RNA-seq, and qRT-PCR assays (Fig. 7A, B and H). Our results suggest that IFN-mediated induction of antiviral host defense genes is not how HAE curb MeV cell-to-cell spread and the growth of infectious centers. However, we see evidence of modestly increased mRNA levels of innate immunity genes in the absence of IFN expression. These data suggest that IFN-independent gene pathways may influence the antiviral state of MeV-infected HAE and contribute to the arrest of MeV cell-to-cell spread.

## DISCUSSION

Modeling MeV infection in HAE has explained previously unknown mechanisms of the rapid MeV spread in human tracheal and bronchial epithelial cells. For example, the observation that MeV infects HAE from the basolateral surface has led to the discovery of its epithelial receptor, the adherens junction protein nectin-4 (22, 52). From there, direct cell-to-cell MeV spread remains nectin-4 dependent (11, 23, 52). The virus forms infectious centers without detectable cytopathic effect, and the infectious centers ultimately detach *en masse* (46). We reproducibly observe that little cell-to-cell MeV spread occurs beyond 3–5 dpi. HAE provide a robust model of *in vivo* innate immune responses to viral and bacterial pathogens. As such, IFN signaling would have been a straightforward explanation to the containment of virus spread in HAE.

Here, we demonstrated that exogenous type I and III IFNs can limit infectious center growth. However, blocking the IFN signaling cascade had little effect on viral spread. To understand the level of IFN produced during infection, we assessed IFN mRNA transcripts and protein levels and found that MeV-infected HAE produced marginal levels of IFN transcripts or proteins, consistent with primate infection studies (36). Likewise, we observed that V^KO^ MeV had no spread defect in HAE. Indeed, we observed a modest but statistically significant increase in V^KO^ MeV infectious center size at 4 dpi as compared to WT MeV. This result suggests that the V^KO^ cell-to-cell spread kinetics is modestly accelerated, but the average final infectious center area is similar to WT MeV. The mechanism for this observation is unknown; however, we postulate that it may be related to the resultant increase in *P* expression when *V* is knocked out (47).

While this study does not answer the question of why infectious center growth is limited, it does rule out the simplest explanation. We acknowledge that despite our efforts at detecting IFN production, some IFN below the limit of detection may contribute to limiting infectious center size; however, we are confident that IFN signaling does not fully explain why MeV cell-to-cell spread stops. In response to MeV infection, we observed increased mRNA levels from multiple genes classically termed interferon-stimulated genes (ISGs). However, in this context, ISG would be a misnomer. The increased mRNA levels of these antiviral genes without IFN suggest that MeV induces a similar response through IFN-independent pathways. Such pathways may contribute to limiting cell-to-cell spread and infectious center growth. Epithelial mesenchymal transition (EMT), cGAS/STING, and/or NF-κB-induced gene expression are potential explanations. EMT was recently shown to stimulate genes normally under the control of IFN such as ISG15, CXCL10, and IFIH1, three genes with MeV-induced mRNA levels (53). Shirogane and colleagues previously showed that induction of EMT renders polarized epithelial cells resistant to MeV infection (54). In addition, we observed increased levels of mitophagy-associated transcripts. The vaccine strain of MeV was found to trigger p62-mediated mitophagy (55). As a result, cGAS targets cytoplasmic mitochondrial DNA released during mitochondrial stress leading to STING-mediated antiviral gene induction (56, 57). Indeed, a cGAS response to MeV was recently demonstrated in Vero-hSLAM and MCF7 cells (58). NF-κB is also well known to induce non-canonical ISG expression (59, 60). Potentially, cGAS/STING, EMT, and/or NF-κB are activated during MeV infection and driving the expression of antiviral gene transcripts. The extent of involvement of all mentioned pathways in response to MeV-infected HAE is currently under investigation.

A likely reason why MeV, and other related paramyxoviruses and pneumoviruses, can gain control over the host IFN pathway is by expressing proteins responsible for antagonizing host IFN specifically (61 – 67). Despite expression of IFN combative proteins, related viruses still induce high levels of IFN in cells compared to our findings in this study (43, 68 – 70). Thus, the question remains of how MeV is so much more contagious than related viruses containing almost identical genomes (1). We believe that the lack of IFN induction in airway epithelia in response to infection may be a link for this phenomenon.

Of note, there is little published information showing head-to-head comparisons of apical versus basolateral release of antiviral cytokines in air-liquid interface models. For the sake of convenience, cytokines are often measured in basolateral media. Our results support the notion that sampling the basolateral media for antiviral cytokine secretion is appropriate.

While we recognize the strengths of an HAE model system, this model has limitations. Though HAE have an intact cellular immune system and retain the ability to secrete cytokines, HAE lack both innate immune cells present from the airways, including natural killer cells, pulmonary alveolar macrophages, and dendritic cells, and the adaptive arm of the immune response. Indeed, we did observe elevated levels of the chemoattractant CXCL10 and pro-inflammatory IL-6, both essential cytokines for initiating migration of several innate and adaptive immune cells and responding to viral infection, respectively (71, 72). Although it may be possible that innate and adaptive immune cells could contribute to halting MeV spread, we note that infectious center growth stops in HAE in the absence of these cells. The role of immune cells in this model system during MeV infection has yet to be explored.

MeV is extremely contagious and consequently nearly impossible to eradicate using current strategies. We know a great deal about how MeV infects and spreads in its human host, but fundamental questions remain about its biology. This is due, in part, to the fact that humans are the only natural reservoir for MeV and representative model systems are challenging to produce and maintain. Within the airway epithelium, MeV undergoes its final amplification and prepares itself for spread to the next host. This final step is likely to contribute to the contagious nature of MeV. Our current working timeline postulates that after the initial infection of airway epithelial cells, the virus spreads cell-to-cell over the next 3–5 days and forms an infectious center. To facilitate viral spread, viral proteins disrupt cytoskeletal networks. As a result, adhesion along the infectious center periphery is lost, and eventually, it is expelled from the epithelial layer. MeV-loaded infectious centers are then projected into the environment *en masse* through coughing and sneezing. This MeV-laden cellular debris would directly deliver the virus to the next host. This study may have applications toward understanding the mechanism of the spread of a significant global health concern.

## MATERIALS AND METHODS

### Human airway epithelial cells

The University of Iowa *In Vitro* Models and Cell Culture Core cultured and maintained HAE as previously described (73). In brief, epithelial cells were dissociated from tracheal and bronchial tissue and seeded onto collagen-coated, semipermeable polycarbonate membranes with a pore size of 0.4 µm (surface area = 0.6 cm^2^; Corning Costar, Cambridge, MA). HAE remained submerged in Ultroser G (USG) medium for 24 h at 37°C and 5% CO_2_. After 24 h, the apical USG medium was removed, and the cells were incubated at the air-liquid interface. All cultures used for experiments described were >3 weeks old and maintained a high transepithelial electrical resistance of 500 Ω μm^2^ or greater.

### Immortalized cells

Vero-hSLAM cells (74) (Vero cells [ATCC cat# CCL-81] with stable expression of human SLAMF1 receptor) were maintained in Dulbecco’s high-glucose modified Eagle’s medium (DMEM; Thermo Fisher Scientific) supplemented with 10% (vol/vol) fetal bovine serum (Thermo Fisher Scientific), 1% penicillin-streptomycin (Thermo Fisher Scientific), amphotericin-B (working concentration of 0.25 µg/mL, Thermo Fisher Scientific), and G418-geneticin (working concentration of 500 µg/mL). H358 cells (bronchioalveolar carcinoma cell line, ATCC cat# CRL-5807) were maintained in Roswell Park Memorial Institute 1640 medium (RPMI 1640; Thermo Fisher Scientific) supplemented with 10% (vol/vol) fetal bovine serum. HEp2 cells (epithelia carcinoma cell line, ATCC cat# CCL-23) were grown and maintained in minimum essential medium (MEM, Thermo Fisher Scientific) supplemented with 10% (vol/vol) fetal bovine serum and 1% penicillin-streptomycin (working concentration of 50 µg/mL–50 U/mL, respectively). THP1 cells (acute monocytic leukemia cell line, ATCC cat# TIB-202) were grown and maintained in RPMI 1640 medium supplemented with 10% (vol/vol) fetal bovine serum and 50 µM 2-mercaptoethanol (VWR Life Science, cat# M131).

### Virus production, propagation, and titer determination

Recombinant MeV expressing GFP or mCherry was generated from infectious MeV cDNAs, isolate Ichinose-B (IC323), rescued, and produced as previously described (7, 75, 76). V^KO^ MeV-GFP was generated as previously described (47). Vero-hSLAM cells were infected with 10 µL of P1 viral stocks for 2 h in Eagle’s minimum essential medium (Opti-MEM, Thermo Fisher Scientific). Fresh supplemented DMEM was placed on cells after infection. Infected Vero-hSLAM lysates were collected at 2–3 dpi and subjected to three freeze/thaw cycles to lyse cells. Lysates were centrifuged to remove cellular debris and supernatants were collected. To determine the titer of MeV, Vero-hSLAM cells were seeded at a density of 10,000 cells/well in a 96-well flat-bottom plate (tissue-culture treated, Corning). Virus stocks were serially diluted and plated onto Vero-hSLAM cells. TCID_50_ titers were determined 3 days later by the presence of cytopathic effect-positive wells. GFP expressing recombinant respiratory syncytial virus (A2 strain) was originally generated by Mark Peeples (77) and was provided to us by Dr. Steven Varga (St. Jude Children’s Research Hospital, Memphis, TN, USA). To generate RSV-GFP stock, HEp2 cells were infected with RSV at an MOI of 1 for 2 h. Five days post infection, RSV-infected HEp2 cell lysates were collected and sonicated to lyse cells. Lysates were centrifuged to remove cellular debris, and supernatants were collected. Titer was determined via TCID_50_ on Vero cells.

### Infection of HAE and immortalized cells

MeV infection of HAE was performed as previously described (22, 23). In brief, and due to the MeV epithelial receptor being located on the basolateral side of the epithelium, HAE cultures were inverted to expose basolateral surface where the MeV inoculum was placed (Fig. S1). Virus was diluted in 50 µL Opti-MEM. Inverted cultures were incubated for 4 h at 37°C under 5% CO_2_. After infection, virus was removed and cultures were returned upright. To infect HAE with RSV, RSV was diluted with Opti-MEM in 50 µL and placed onto apical surface for 4 h under the same conditions as indicated above. After 4 h, virus was removed and cultures were rinsed three times with Dulbecco’s phosphate-buffered saline, no calcium, no magnesium (DPBS−/−, Thermo Fisher Scientific). All experiments included cultures from at least three separate human donors. Vero-hSLAM or H358 cells were plated onto 48-well tissue culture-treated plates (Corning) at a density of 30,000 cells/well and incubated overnight. Cells were infected at an MOI of 0.1 for 2 h in Opti-MEM. Virus inoculum was removed, and cultures were gently washed three times with DPBS−/− before replacing culture medium.

### LDH-cytotoxicity colorimetric assay

Supernatants from infected cultures were collected daily followed by a gentle wash with DPBS−/−. Fresh medium was replaced on each culture. LDH was measured following manufacturer’s protocol (BioVision, cat# K311). In brief, 100 µL of reaction mixture was combined with 100 µL of supernatant in a 96-well Maxi-Sorp Immunoplate (Thermo Fisher Scientific). Mixture was incubated at room temperature in the dark for 30 min. Wells containing sample were read for absorbance at 490_OD_. To calculate cytotoxicity, the following formula was used: % cytotoxicity = (test sample – low control) ÷ (high control – low control). Low controls were mock-infected supernatants at each given time point. To obtain high controls, cells were incubated with media containing 0.1% Triton X-100 for 30 min to induce complete cell death.

### Immunostaining and microscopy

Infected HAE were fixed with 2% paraformaldehyde for 15 min and blocked and permeabilized in Pierce SuperBlock (Thermo Fisher Scientific) containing 0.1% Triton X-100 at room temperature for 1 h. Cultures were then incubated with rabbit anti-N_505_ (1:200) (23) overnight at 4°C. Secondary antibody 1:10,000 goat anti-rabbit was incubated with cultures in permeabilization buffer at room temperature for 1 h. Transwell filters containing the fixed and stained cells were cut from the plastic transwell and mounted onto glass slides. VECTASHIELD Mounting Medium containing DAPI (4′,6′-diamidino-2-phenylindole; cat# H-1200–10, Vector Laboratories, Inc., Burlingame, CA) was applied directly to the filter. Glass coverslips were added and sealed with clear nail polish. All confocal images were captured on a Leica TCS SPE confocal microscope (Leica Microsystems, Inc.) using a 40× oil immersion objective. Images were processed using ImageJ version 2.3.0/1.53q. Cell images were captured using a Leica Fully Automated Inverted Research Microscope DMI 6000 B (Leica Microsystems, Inc.) with a 2.5× objective or with an All-in-one-Fluorescence Microscope BZ-X800E (Keyence Corporation of America, Itasca, IL, USA) as noted in figure legends.

### Infectious center area measurements

All infectious center areas were measured using ImageJ version 2.3.0/1.53q and images were blinded using the blind-analysis plug-in tool. Images were loaded and subjected to the color threshold tool. All images were then converted to 8-bit and processed using the convert to mask and watershed processing tools. Particles were then analyzed for area, and data were put into excel files and unblinded.

### RSV fluorescence intensity measurements

All RSV images were blinded and measured identical to the methods listed above. Images were loaded and subjected to intensity density measurements. Increased infection was commonly observed along the periphery of the culture. This “edge effect” likely occurs due to a combination of lack of tight junctions where the cells meet the plastic and the fluid meniscus while the virus is applied to the apical surface. The periphery was cropped from the images prior to data analysis. All data were collected, processed, and unblinded in excel. Once unblinded, fluorescence intensity was calculated as follows: fluorescence intensity (% mock treated) = (test sample – uninfected control) ÷ (mock-treated control – uninfected control).

### Interferon treatment and blocking

MeV-infected HAE were treated apically with 100 ng/mL recombinant human IFN-β (StemCell Technologies cat# 78113.1) or 2 µg/mL IFN-λ1 (Sigma-Aldrich, St. Louis, MO cat# SRP3059) 18 hpi. Twenty-four hours post application, treatments were removed and cultures were gently washed with DPBS three times. To induce IFN-blocking, MeV-infected HAE cultures were treated apically with 2 µg/mL recombinant B18R protein (StemCell Technologies cat# 78075) or 50 ng/mL monoclonal anti-human IFN-λ1 neutralizing antibody (clone MMHLR1, PBL Assay Science, Piscataway, NJ, cat# 21885-1).

### Quantitative reverse transcriptase polymerase chain reaction

At indicated timepoints, TRIzol reagent (Thermo Fisher Scientific cat# 15596026) was added to the apical surface of cells. Filters were scraped gently with a pipette tip and cells were collected. RNA was isolated from cells using the Direct-zol RNA Miniprep kit (Zymo Research cat# R2052). RNA was converted to cDNA using High-Capacity RNA-to-cDNA kit (Thermo Fisher Scientific cat# 4387406). PCR was performed targeting indicated genes and quantified compared to the reference gene *SFRS9*. See Table S1 for primer pairs.

### Western blot

Vero-hSLAM cells were mock infected or infected with WT-MeV-mCherry or V^KO^-MeV-GFP at an MOI of 0.01 for 2 h. Cells were lysed with RIPA Lysis and Extraction Buffer (Thermo Fisher Scientific cat# 89900). Lysis buffer was supplemented with cOmplete, Mini EDTA-free Protease Inhibitor Cocktail (Millipore Sigma, Darmstadt, Germany cat# 11836170001). Protein concentrations were determined using the Pierce BCA Protein Assay Kit (Thermo Fisher Scientific cat# 23225). Laemmli buffer was added, and each sample was boiled at 95°C for 5 min. Twenty-five micrograms of each protein sample was loaded into a 4%–20% Mini-PROTEAN TGX Precast Protein Gel (BioRad, Hercules, CA cat# 4561094). Protein was run on gels at 100 V for 30–45 min. Gels were transferred onto PVDF membranes at 25 mA overnight at 4°C. Membranes were blocked with 1× TBS buffer supplemented with 0.1% Tween and 5% milk for 1 h at room temperature. Primary polyclonal antibodies against the C-terminus region of V protein 6, monoclonal rabbit anti-vinculin (Thermo Fisher Scientific cat# 700062), and polyclonal rabbit anti-N_505_ were all used at a 1:1,000 dilution. For the secondary antibody, horseradish peroxidase-conjugated goat anti-rabbit (Millipore Sigma cat# 111-035-144) was used at 1:10,000 dilution for 1 h. Blots were developed with SuperSignal West Pico PLUS Chemiluminescent Substrate (Thermo Fisher Scientific cat# 34580).

### LEGENDplex human antivirus response panel (13-plex) cytokine screen

Apical washes and basolateral medium were collected from infected HAE at indicated time points. Samples were subjected to the LEGENDplex Human Anti-Virus Response Panel (13-plex) (Biolegend, V-bottom plate cat# 740390). To describe the assay in brief, collected samples were incubated with specific capture-antibody-coated beads detecting 13 antiviral cytokines including IFN-α2, IFN-β, IFN-λ1, IFN-λ2/3, IFN-γ, IL-6, CXCL10, TNF-α, IL-8, IL-10, IL-12p70, GM-CSF, and IL-1β. Sample and beads were rocked at room temperature for 2 h. After the capture beads were washed, biotinylated antibodies were added and incubated with the capture beads for an additional hour. Lastly, phycoerythrin-conjugated streptavidin was added. Cytokine was quantified using flow cytometry and analyzed with the provided LEGENDplex analysis software (Qognit, Inc.).

### Differentiation and infection of THP1 cells

THP1 cells were grown and differentiated as previously described (78). Briefly, cells were grown in suspension at a density range of 300,000 to 800,000 cells/mL and were not passaged more than eight times. To differentiate THP1 cells into macrophage-like cells, cells were seeded onto 12-well plates at a density of 500,000 cells/well in the presence of phorbol 12-myristate 12-acetate (PMA, working concentration of 100 ng/mL, R&D systems cat# 1201) for 2 days. Media containing PMA was removed, and cells were washed three times with HBSS. Fresh media were placed on cells, and cells rested for 24 h prior to infection. After 24 h, cells were infected with MeV-mCherry or MeV V^KO^-GFP at an MOI of 1 in Opti-MEM for 4 h. Virus inoculum was removed, and cells were washed three times with HBSS before replacing media on cells.

### Flow cytometry preparation and analysis of infected THP1 cells

At indicated time points post infection, THP1 cells were stained with 1:1,000 LIVE/DEAD Near-IR Dead Stain (Thermo Fisher Scientific cat # L34976) for 15 min in the dark. Cells were then lifted with Accutase (Innovative cell technologies cat# AT 104) for 10 min. After lifting, cells were spun down and washed with DPBS and then fixed with 4% paraformaldehyde for 15 min. Cells were spun down and washed with DPBS once again. Final cell pellet was resuspended in 500 µL of DPBS and subjected to flow cytometric analysis. Cells were gated for live cells and then further for mCherry or GFP.

### MeV infection and RNA Seq analysis

For the RNA seq experiment, HAE were infected with MeV-GFP at an MOI of 1 for 4 h at 37 °C 5% CO_2_. Total RNA was extracted from infected HAE cultures at 0, 12, 24, 48, 72, and 120 hpi using RNeasy Mini kit (Qiagen) according to manufacturer’s instructions. RNA samples were treated with RQ1 RNAse-Free DNAse for 10–15 min at 37°C to remove genomic DNA contamination. The concentration and purity of the RNA were detected using a Nanodrop 2000 (Thermo Fisher Scientific, USA) and integrity was assessed via RNA integrity number (RIN) generated by capillary electrophoresis. Samples with RNA quantification ≥500 ng and an RIN ≥8 were used to generate Illumina-sequencing libraries. The cDNA libraries were prepared using the TruSeq RNA sample Prep (Illumina, San Diego, CA, USA) according to manufacturer’s instructions and submitted to Iowa Institute of Human Genetics Genomics Division at University of Iowa for sequencing. Adapter sequences and low-quality reads were discarded from the data set. Remaining reads were aligned to the human genome and normalized using RPKM method.

### Statistical analyses

Unless stated otherwise, each study was performed with at least three donors. Statistical analyses are specified in each figure legend. Significant values are shown by asterisks: *P < 0.05; **P < 0.01; ***P < 0.001; ****P < 0.0001 or ns, not significant.

## Data Availability

Single cell RNA sequencing data were previously described (46) and deposited in the GEO database with accession number GSE168775. The bulk RNA sequencing data were deposited in the GEO database with accession number GSE229685.

## SUPPLEMENTAL MATERIAL

Supplemental material spectrum.01361-23-s0001.pdf Fig. S1 to S8 and Table S1.

ASM does not own the copyrights to Supplemental Material that may be linked to, or accessed through, an article. The authors have granted ASM a non-exclusive, world-wide license to publish the Supplemental Material files. Please contact the corresponding author directly for reuse.
