# Supplementary material for: Interferon-independent processes constrain measles virus cell-to-cell spread in primary human airway epithelial cells
Source: Microbiol Spectr. 2023 Sep 19;11(5):e01361-23. doi: 10.1128/spectrum.01361-23 (PMC10580916; doi:10.1128/spectrum.01361-23)
Supplement: Supplemental material — Fig. S1 to S8 and Table S1. [file spectrum.01361-23-s0001.pdf]

Supplemental Figure 1

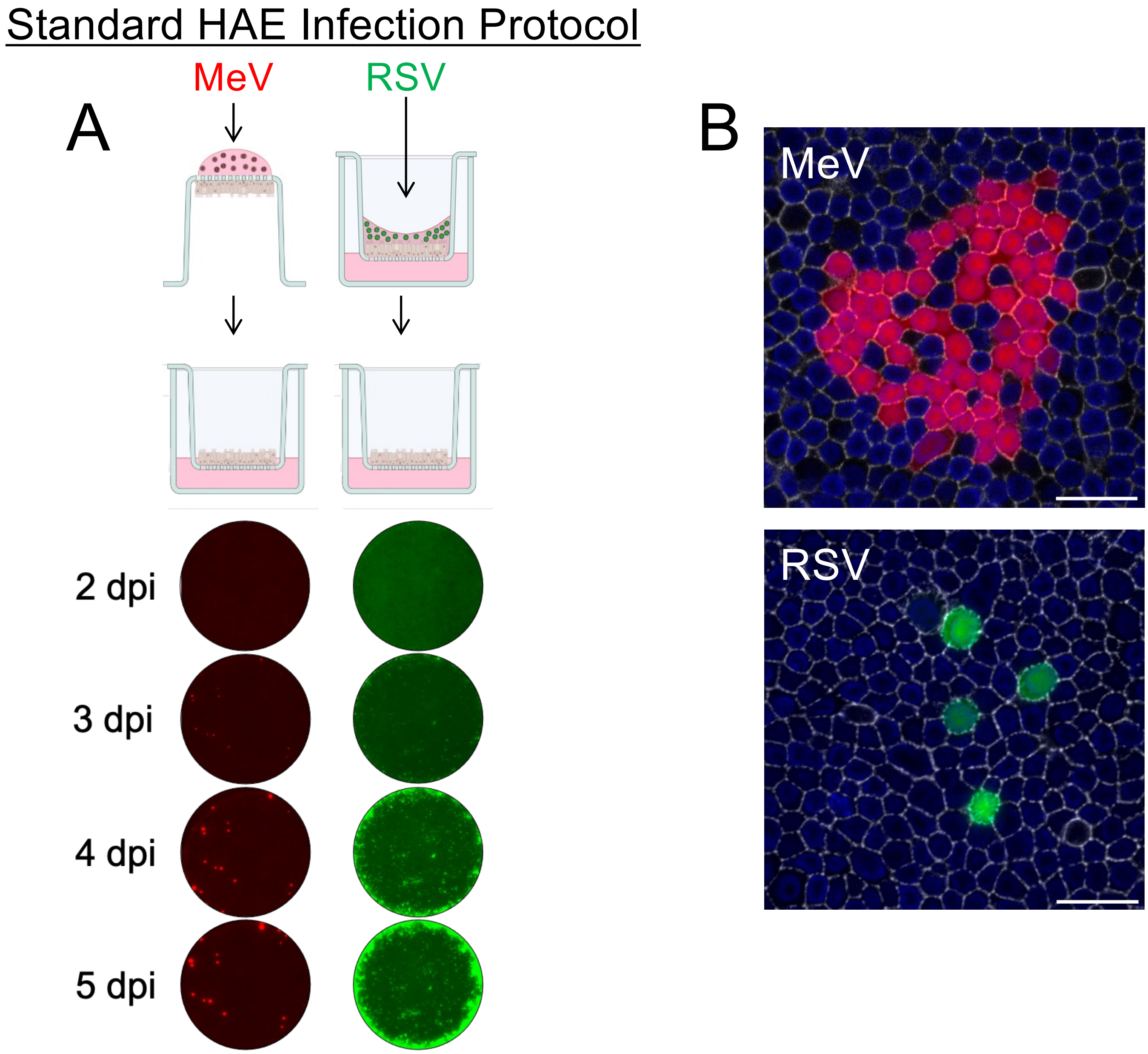

Supplemental Figure 1: Standard infection protocol of human airway epithelial cells (HAE). To infect with WT MeV (mCherry reporter), the basolateral side of each HAE culture was exposed by inverting the transwell. Virus inoculum was placed on the basolateral surface in a volume of 50 μL. After 4 hours, virus inoculum was removed and the cultures were flipped back to an upright position and incubated until the conclusion of the experiment. To infect with RSV (GFP reporter), virus inoculum was placed directly onto the apical surface of HAE for 4 hours. After the infection period, the inoculum was removed and the cells were gently washed with PBS. Low power images were collected daily for 5 days using a Keyence fluorescence microscope. Scale bar = 500 μm. Created with BioRender.com. (B) MeV- or RSV-infected cultures were fixed at 3 dpi for confocal analysis. Top image shows an mCherry+ MeV infectious center. Bottom image shows a GFP+ RSV infected cells. Blue: DAPI, grey: F-actin. Scale bar = 25 μm.

Supplemental Figure 2

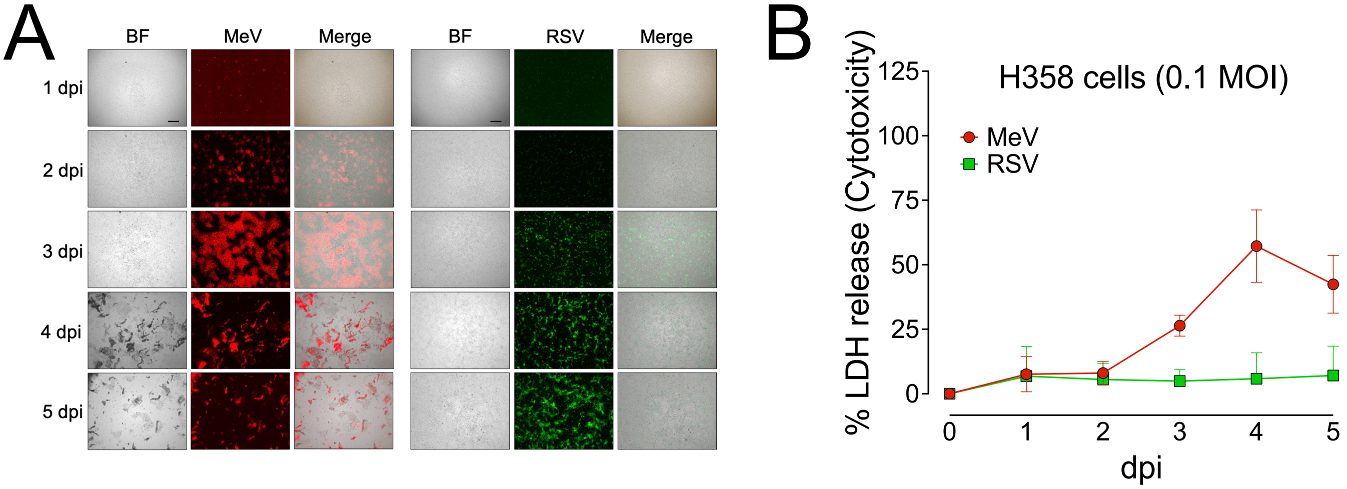

Supplementary Figure 2: MeV induces cell death in H358 cells.
(A) H358 cells 1-5 days post-infection are shown. Infected cells were imaged on a Keyence fluorescence microscope. Brightfield (BF), reporter virus fluorescence, and merged images are shown. Scale bar = 3 mm. LDH cytotoxicity levels from the supernatants of MeV or RSV (0.1 MeV) infected (B) H358 cells plated at 50,000 cells/well and infected for 2 hours. n = 3 wells per virus infection. Data are plotted as mean ± SE.

Supplemental Figure 3

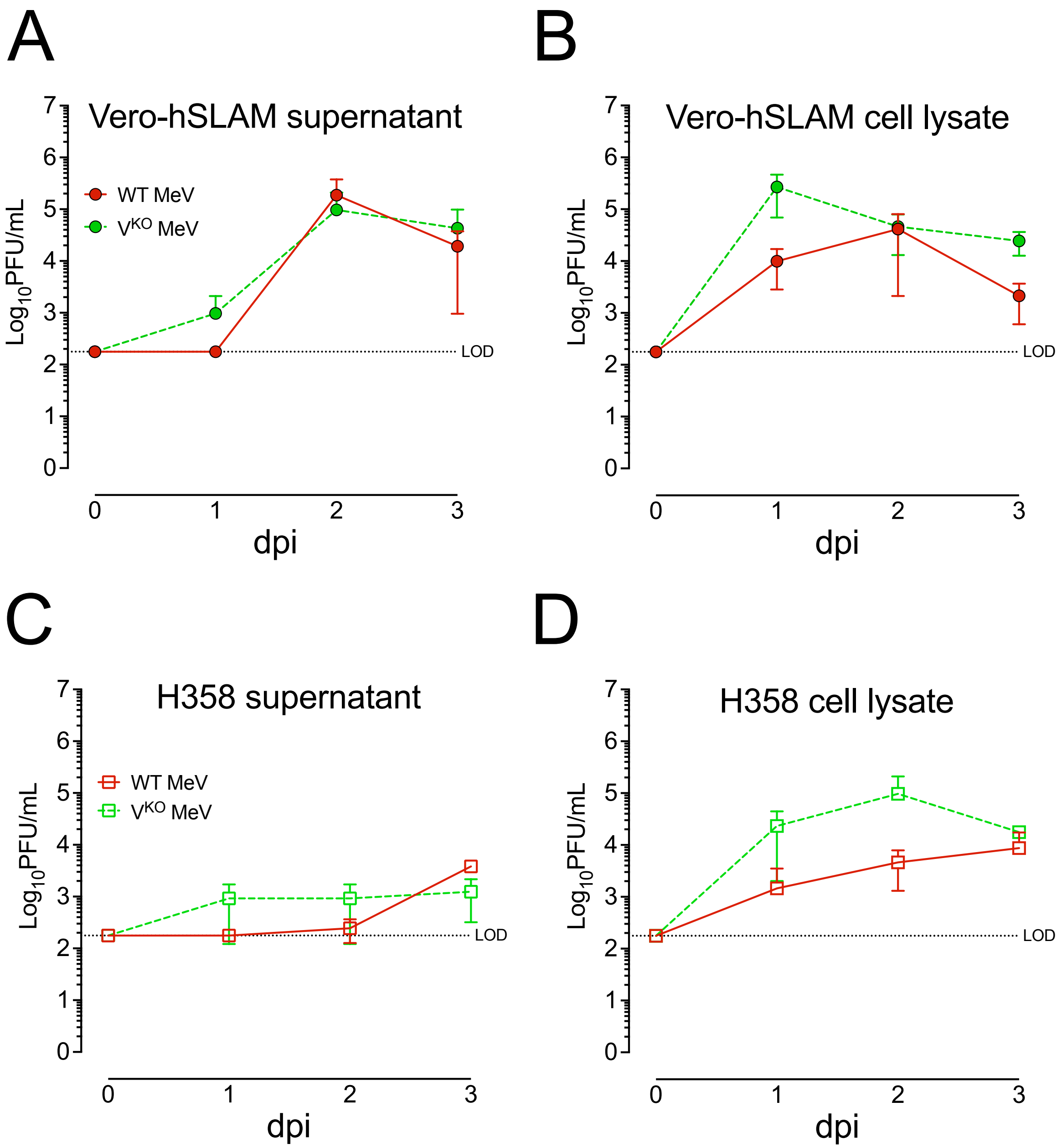

Supplementary Figure 3: V^{KO} MeV growth is not impaired compared to WT MeV.
(A and B) Vero-hSLAM and (C and D) H358 cells were infected (0.1 MO1) of WT MeV (red) or V^{KO} MeV (green) for 2 hours in serum-free medium. (A and C) Supernatants and (B and D) cell lysates were collected daily for 3 days post-infection (dpi). Cell lysates were subjected to three rounds of freeze-thaw cycles to lyse cells. Lysates were centrifuged to remove cellular debris. Supernatants and clarified cell lysates were titrated on Vero-hSLAM cells using a $TCID_{50}$ assay.

Supplemental Figure 4

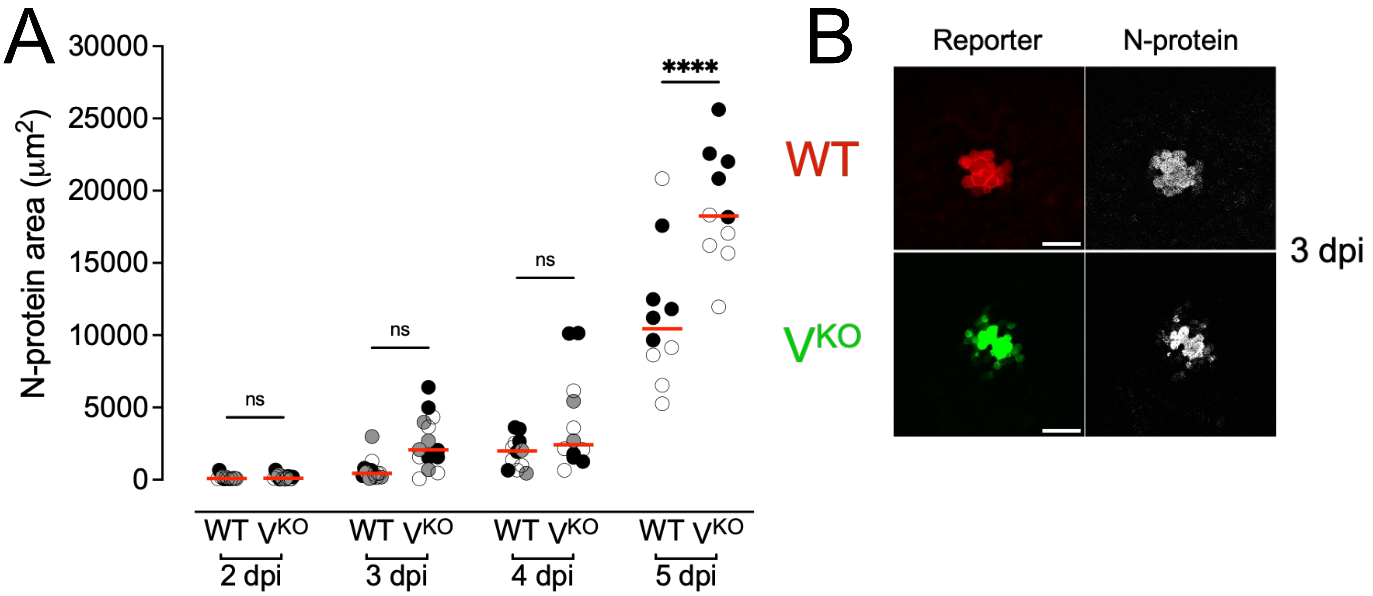

Supplementary Figure 4: Viral antigen immunocytochemistry confirms that V^{KO} MeV infectious centers are not smaller than WT.
HAE were infected with WT MeV or V^{KO} MeV (1 MOI) for 4 hours. At indicated timepoints, immunocytochemistry was used to detect N-protein of co-infected cultures. Images were blinded in gray scale in ImageJ prior to measurements using a blind-analysis tool. Following area measurements, color was unblinded prior to GraphPad Prism analysis. n = 3 donors, indicated by color. Significance was determined by one-way ANOVA with Tukey's multiple comparison corrections. (B) Representative images are shown. Scale bar = 50 μm.

Supplemental Figure 5

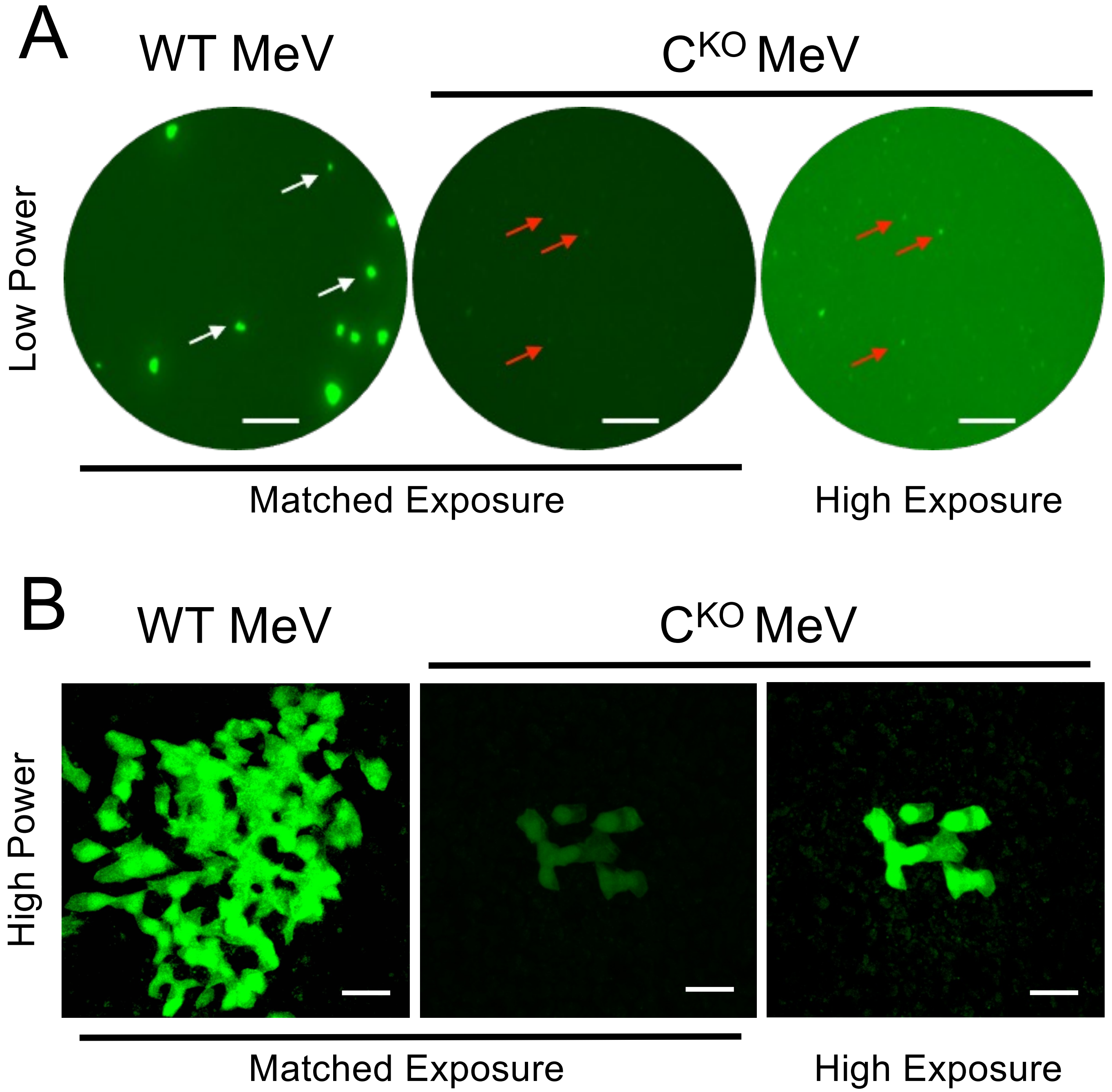

Supplementary Figure 5: C^{KO} MeV infectious centers are small and faint.
HAE were infected with WT MeV or C^{KO} MeV (1 MOI) for 4 hours. (A) WT- or C^{KO}-infected cultures were imaged at low, matched exposures (left two images) at 3 dpi. Far right image shows a C^{KO}-infected culture at a higher exposure to illustrate faint florescence. White arrows point to WT infectious centers. Red arrows point to the C^{KO} infectious centers. Scale bars = 200 μm. (B) WT and C^{KO}-infected cultures were fixed at 3 dpi. Left two images were captured at low, matched exposures (Far left: WT, middle: C^{KO}). Far right image shows the same C^{KO} infectious center at a higher exposure. Scale bars = 20 μm.

Supplemental Figure 6

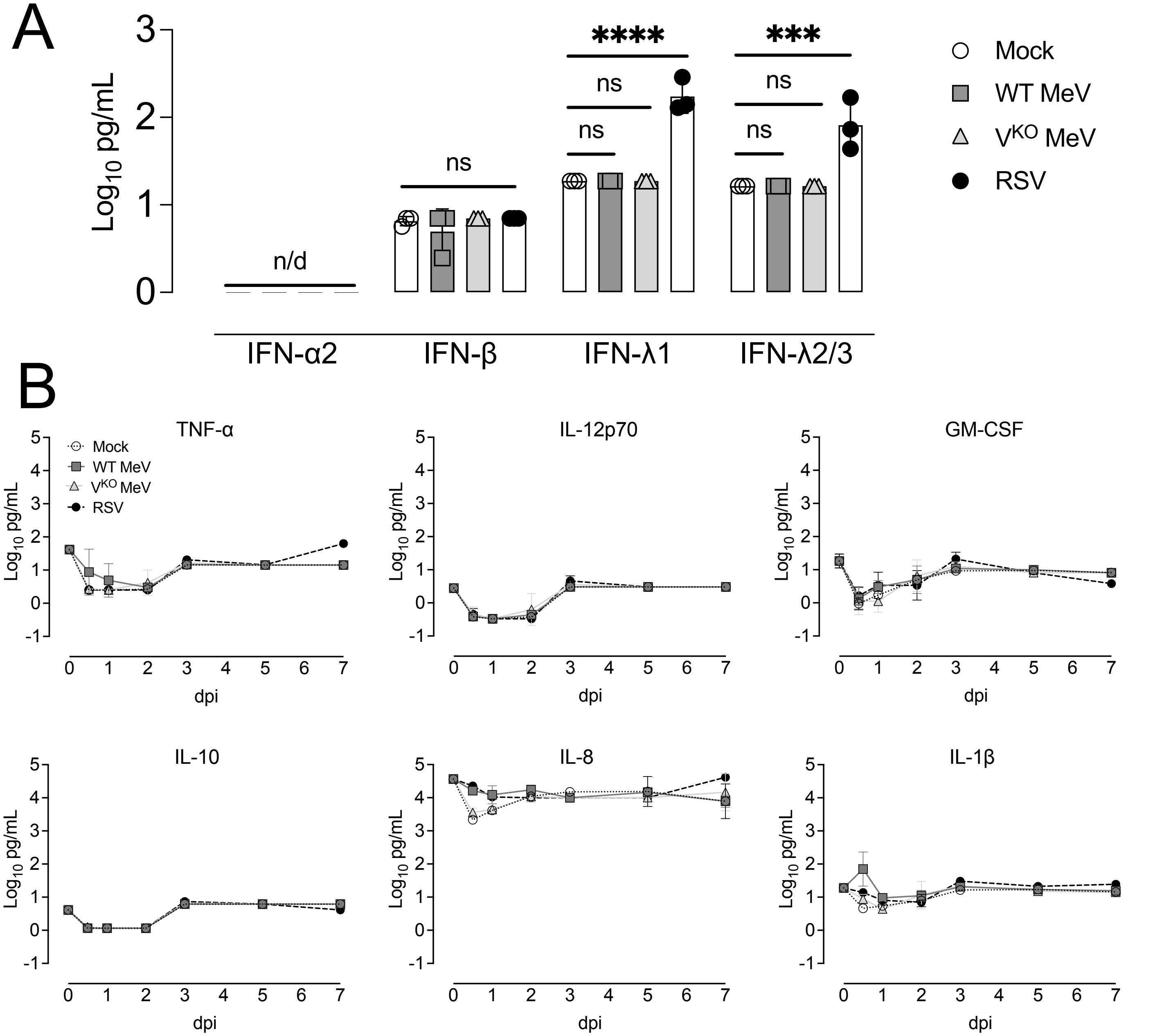

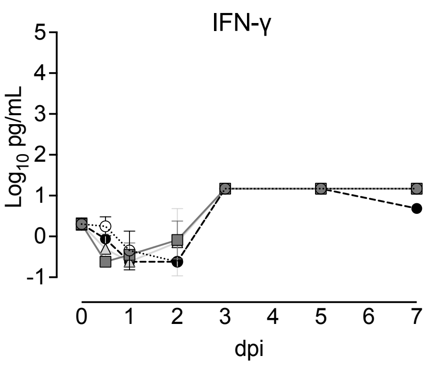

Supplementary Figure 6: Neither WT MeV nor V^{KO} MeV induces selected inflammatory cytokines or IFN after five days of accumulation. A) HAE were either mock infected or infected with WT MeV, V^{KO} MeV, or RSV (1 MOI) for 4 hours. Basolateral media were collected 5 days after infection. Samples were analyzed for presence of IFN-α2, IFN-β, IFN-λ1, and IFN-λ2/3. B) HAE were either mock infected or infected with RSV, WT MeV or V^{KO} MeV (1 MOI) for 4 hours. Basolateral media were collected 12 hours post-infection and everyday up to 7 days post-infection. Basolateral medium was replenished for each culture with fresh media after collection. Samples were analyzed for presence of TNF-α, IL-12p70, GM-CSF, IL-10, IL-8, IL-1β, IFN-γ using a human antiviral response panel. Significance was determined by one-way ANOVA with Tukey's multiple comparison corrections but no significant differences were observed. RSV, solid black circles, dashed black line; WT MeV, solid gray squares, solid gray line; V^{KO} MeV solid light gray triangles, light gray line; mock infected, open circles, black dotted line.

Supplemental Figure 7

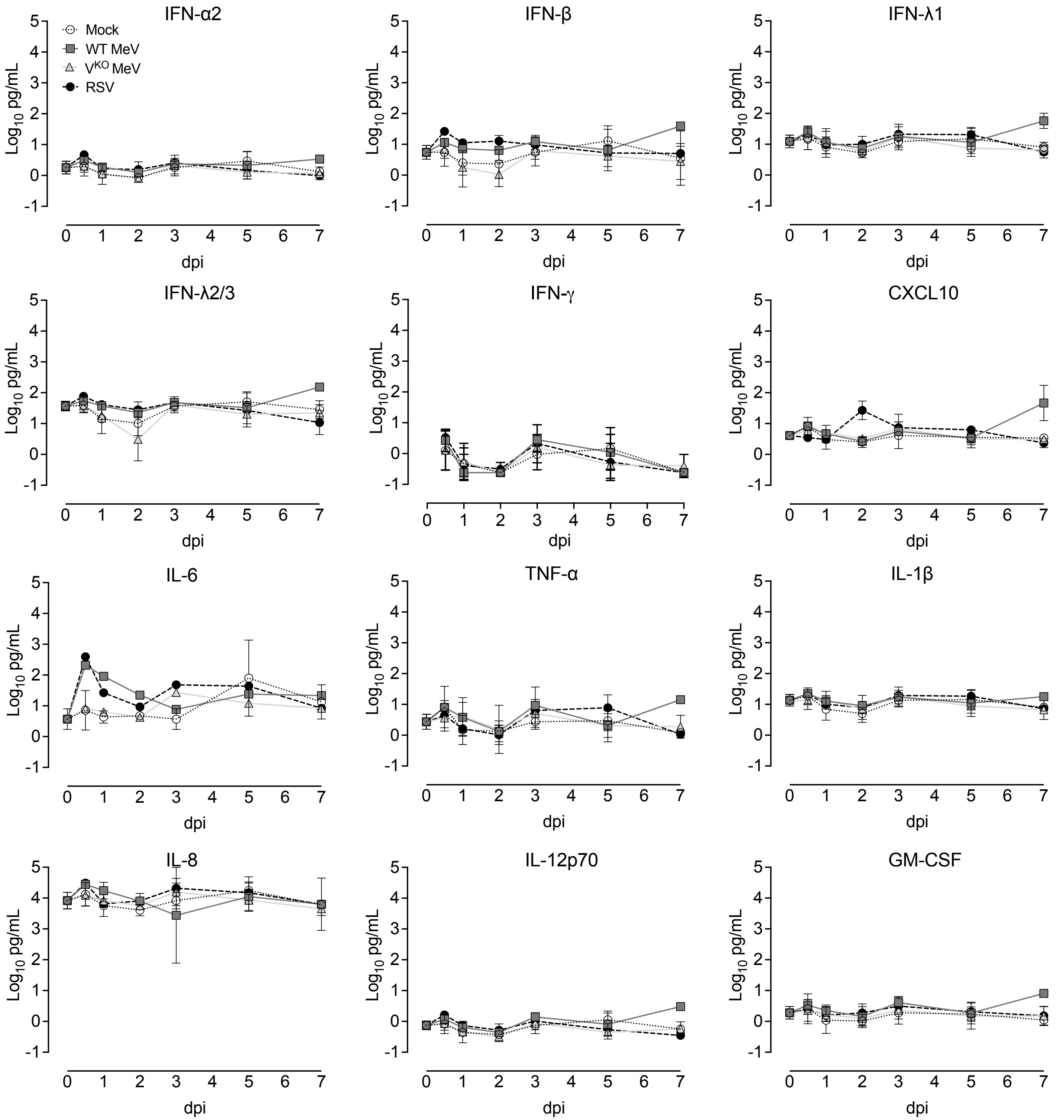

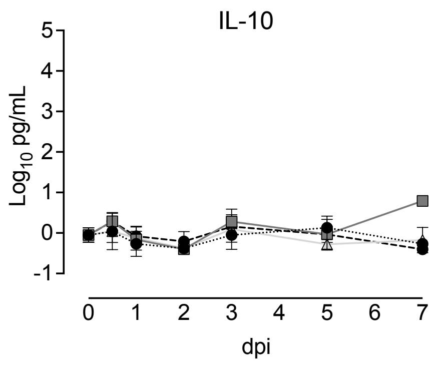

Supplementary Figure 7: Neither WT MeV nor V^{KO} MeV induces apical release of inflammatory cytokines.
HAE were either mock infected or infected with RSV, WT MeV or V^{KO} MeV (1 MOI) for 4 hours. Apical washings were collected 12 hours post-infection and everyday up to 7 days post-infection. Samples were analyzed for presence of IFN-α2, IFN-β, IFN-λ1, IFN-λ2/3, TNF-α, CXCL10, IL-6, IL-1β, IL-12p70, GM-CSF, IL-10, IL-8, and IFN-γ using a human antiviral response panel. Significance was determined by one-way ANOVA with Tukey's multiple comparison corrections but no significant differences were observed. RSV, solid black circles, dashed black line; WT MeV, solid gray squares, solid gray line; V^{KO} MeV solid light gray triangles, light gray line; mock infected, open circles, black dotted line.

Supplemental Figure 8

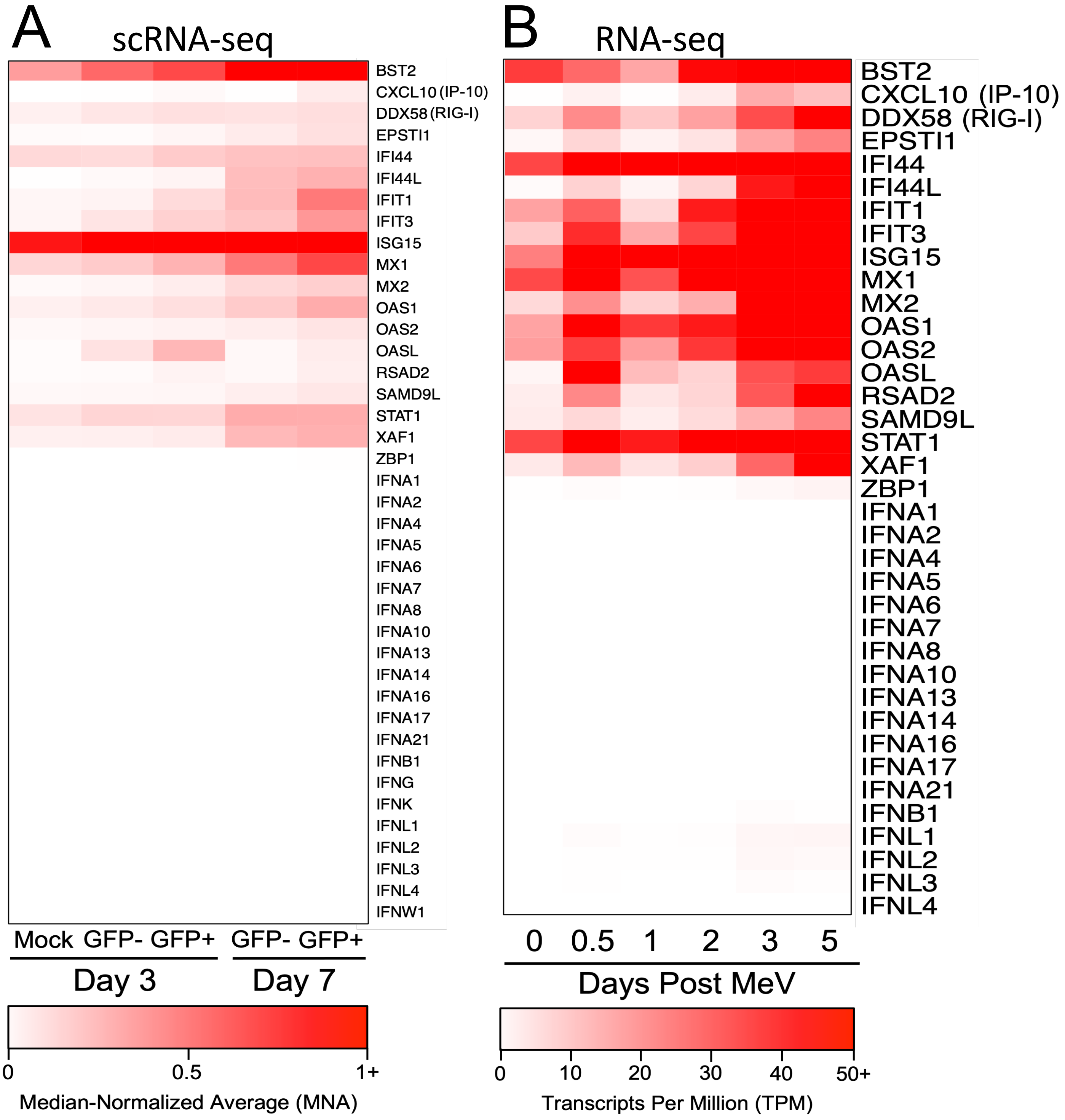

Supplemental Figure 8: No IFN RNAs present in the bioinformatic panel are elevated during MeV infection of HAE.

(A) Single-cell RNA sequencing was performed on mock or MeV-infected cultures 3- and 7-days post-infection. Each MeV infected culture was FACS sorted into GFP+ or GFP- populations. Intensity shows measure of raw gene transcript counts normalized for the number of cells and total transcript reads in each group (MNA). Every condition included 10 pooled, matched donor HAE cultures and sequencing was performed with a 10x Genomics scRNA-seq. (B) A heat map is shown depicting expression levels for selected innate immune genes measured by RNAseq of MeV-infected HAE. Transcript abundances are expressed as transcripts per million (TPM). MNA and TPM are both measures of raw transcript reads; however, direct comparison of the color intensity between the two heat maps is not necessarily meaningful. HAE were infected with MeV and collected for sequencing at 0, 0.5, 1, 2, 3, and 5 dpi. n =4 donors. Heatmaps were constructed using RStudio.

Gene	Direction	Sequence
IFN-β	FWD	5'GTTGAGAACCTCCTGGCTAATG3'
	REV	5'GGTAATGCAGAATCCTCCCATAATA3'
IFN-λ1	FWD	5'ACATCCACGTCGAACTTCAGGCTT3'
	REV	5'GCTTGAGTGACTCTTCCAAGGC3'
CXCL10	FWD	5'TGTCCACGTGTTGAGATCATTGC3'
	REV	5'ATTCTTGATGGCCTTCGATTCTGG3'
STAT1	FWD	5'TGCGTAGCTGCTCCTTTGGTT3'
	REV	5'AGTTCGTACCACTGAGACATCCTG3'
STAT2	FWD	5'TCAGGAAAGGGCAGCAATAAGCC3'
	REV	5'ACCGTGAAGCTGATGATGTGCAG3'
IRF3	FWD	5'AATCCCACTCCCTTCCCAAACC3'
	REV	5'TCACCTCGAACTCCCACTCTTC3'
IRF9	FWD	5'AGTCCATTCAGACATTGGGAGCAG3'
	REV	5'GGCCTCAGTTGTGTCTGTAACTTC3'
OAS1	FWD	5'TGAGCTCCTGGATTCTGCTGAC3'
	REV	5'TGGCATTCAGAGGATGGTGCAG3'
MX1	FWD	5'TAACCTCCACAGAACCGCCAAGTC3'
	REV	5'TGAAAGCAAGCCGGACCATATCC3'
RIGI	FWD	5'TGATCCATCTGGCTCCTCACCGTG3'
	REV	5'TTCACCTCTGCACTGTTGCTCAG3'
MDA5	FWD	5'GATTGCTCAGAAAGCAATGCAGAG3'
	REV	5'AAGAAGTTGCTCTTCCACTTGAGG3'
SFRS9	FWD	5'TGCGTAAACTGGATGACACCC3'
	REV	5'CCTGCTTTGGTATGGAGAGTC3'

Supplemental Table 1. Primer pairs used for quantitative reverse transcriptase polymerase chain reaction (qRT-PCR).
